# Quantum Neural Network Realization of XOR on a Desktop Quantum Computer

**DOI:** 10.3390/s26030854

**Published:** 2026-01-28

**Authors:** Tee Hui Teo, Qianrui Lin, Yiyang Fu

**Affiliations:** Singapore University of Technology and Design, Singapore 487372, Singapore; qianrui_lin@mymail.sutd.edu.sg (Q.L.); yiyang_fu@mymail.sutd.edu.sg (Y.F.)

**Keywords:** nuclear magnetic resonance, quantum computer, quantum machine learning, quantum neural networks, variational quantum circuit

## Abstract

Quantum neural networks leverage quantum computing to address machine learning problems beyond the capabilities of classical computing. In this study, we demonstrate a quantum neural network that learns the nonlinear exclusive OR function on a desktop quantum computer. The exclusive OR task is a nonlinear benchmark that cannot be solved by a single-layer perceptron, making it an excellent test for quantum machine learning. We trained a variational quantum circuit model in a simulation using the PennyLane framework to learn the two-bit exclusive OR mapping. After obtaining the circuit parameters in the simulation, the trained quantum neural network was deployed on a two-qubit Nuclear Magnetic Resonance-based desktop quantum computer operating at room temperature to evaluate the actual hardware performance. The experimental quantum state fidelity reached approximately 98.85%$\left(R_{y}\right)$ and 99.35%$\left(R_{x}\right)$, and the overall average purity was 95.16%$\left(R_{y}\right)$ and 97.43%$\left(R_{x}\right)$, indicating excellent agreement between the expected and measured results. These positive outcomes underscore the feasibility of quantum machine learning on small-scale quantum hardware, marking a minimal yet physically meaningful benchmark.

## 1. Introduction

Quantum computing is a novel approach based on the principles of quantum mechanics. It differs from the classical computing method, which uses traditional bits, in that quantum computing employs quantum bits (qubits) to represent and process information. Qubits are fundamental units in quantum computing and can exist in multiple states simultaneously, not just 0 or 1, until their values are determined by measurement [1]. This phenomenon is known as “quantum superposition”. Compared to the binary bits of traditional computers, quantum computers can achieve an exponential leap in solving problems, making them “far ahead” [2].

Quantum computing has opened new ways to solve problems that are difficult for classical computers to handle [3]. One of the most exciting fields is quantum machine learning (QML), which combines the concepts of quantum physics and artificial intelligence (AI) [4]. QML is expected to accelerate tasks such as data analysis and optimization. Quantum computing has been shown to excel in factorization issues and unordered search problems owing to its quantum parallelism capability, which allows for an exponential speedup in solving certain problems [5]. Quantum Neural Networks (QNNs) combine the efficiency of quantum computing and the learning ability of neural networks, providing a new approach for solving complex problems that are difficult for traditional AI to handle. Owing to the superposition and entanglement properties of qubits, QNN can process a large amount of data simultaneously, achieving faster and more accurate results [6].

Why did we choose to use a QNN for the exclusive OR (XOR) problem? The XOR problem is a well-known nonlinear and inseparable problem in classical machine learning [7]. Minsky and Papert (1969) proved that single-layer perceptrons cannot represent non-linearly separable functions such as XOR [8]. Traditional single-layer neural networks, also known as perceptrons, have the characteristic that the input layer is equal to the output layer, with only one weight layer [9], as expressed in Equation (1).

(1) $$\begin{array}{ccc} y & = & f\;\left(w_{1}x_{1}+w_{2}x_{2}+b\right). \end{array}$$

Its decision boundary is a straight line; therefore, it can only separate data linearly. The truth table of XOR is shown in Table 1, where (0,1) and (1,0) belong to the positive class and (0,0) and (1,1) belong to the negative class [10]. If one attempts to separate the positive and negative classes with a straight line in a plane coordinate system, it will be found that no straight line can simultaneously separate these two groups, which is the essence of linear separability. Although XOR is not a basic quantum gate, it is the minimum functional benchmark for verifying whether quantum computing and quantum neural networks possess the ability for nonlinear information processing, and therefore, it holds significant methodological significance in quantum computing architectures and learning models.

However, most current research on QNNs and variational quantum circuits (VQCs) remains in the simulation stage [11,12], with few studies deploying trained QNNs to practical quantum hardware, especially lacking experimental reports based on desktop-level quantum devices at room temperature [13]. Moreover, it is unclear whether small-scale quantum machines can stably execute QNNs and whether their accuracy is sufficient. These research challenges are addressed in this study [14].

In addition to the quantum computing platform, the storage and manipulation of quantum information are also widely present in various physical nanostructures, such as quantum dots, spin systems, optical polarization systems, etc. These physical implementations also reflect the nonlinear characteristics introduced by quantum state manipulation and measurement projection, and have been used to explore the basic mechanisms of information processing and storage [15]. The quantum neural network studied in this paper shares common quantum information processing features at the methodological level with the aforementioned physical systems.

In this study, we trained a 2-qubit VQC in PennyLane [16] to learn the XOR operation and transplanted the obtained parameters into a desktop quantum computer [17]. We ran the four inputs of |00〉, |01〉, |10〉, |11〉 on the real quantum hardware and conducted quantum state tomography, achieving high accuracy (97%) and high fidelity (98.85%). This study demonstrates that small-scale quantum hardware can execute QML models, showcases the robustness of QNNs in real physical systems, proves that migration from simulators to hardware is feasible, and supports future scalable quantum artificial intelligence (AI) applications [18], such as supervised learning assisted by an entangled sensor network (SLAEN) [19]. SLAEN uses a VQC to generate multipartite entangled probe states for distributed sensors, combined with a classical support vector machine (SVM) for data classification. It demonstrates quantum-enhanced performance in classifying radio-frequency (RF) signals, achieving lower error probabilities than classical methods.

Although the XOR task can be easily accomplished through traditional logic circuits or classical neural networks, the purpose of this paper in using quantum neural networks and real quantum hardware to complete this task is not to surpass performance, but to verify the methodology. As XOR is the smallest nonlinear separable problem, it provides a controllable and highly interpretable benchmark for evaluating whether the model has nonlinear expression capabilities. In quantum neural networks, nonlinear mapping does not originate from classical activation functions but is jointly produced by quantum state evolution, entanglement, and measurement processes. Therefore, XOR is an ideal test platform for verifying nonlinear sources. Furthermore, implementing this task on real quantum hardware helps evaluate the physical feasibility and stability of the variational parameters in the presence of continuous noise and measurement errors. Thus, the contribution of this paper lies in demonstrating the minimum feasible learning ability of quantum neural networks on Noisy Intermediate-Scale Quantum (NISQ) hardware, laying an experimental foundation for more complex quantum learning tasks in the future.

This paper is organized as follows: Section 2 details the design and implementation of the VQC, including the circuit architecture, input-output encoding scheme, training procedure, and the transfer of parameters from simulation to hardware. Section 3 describes the experimental environment, the desktop (NMR) quantum computer used as the hardware platform, calibration procedures, and the step-by-step circuit implementation protocol. Section 4 presents the training convergence, optimized parameters, model performance on the XOR truth table, and experimental results, including the quantum state tomography, fidelity, and purity metrics. Finally, Section 5 summarizes the findings and outlines future research directions.

## 2. Methodology

**Figure 1:**
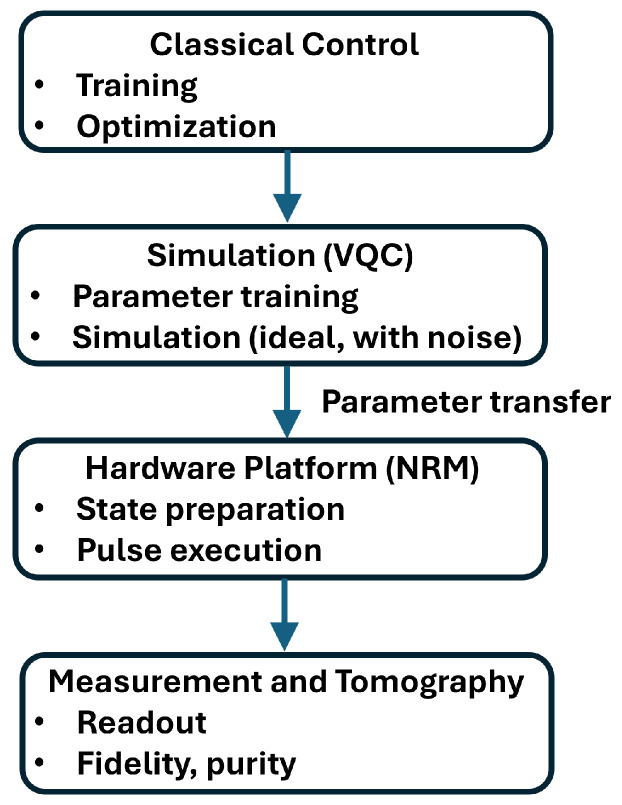
Hardware platform and implementation workflow of the proposed QNN.

The Methodology section outlines the design and implementation of the VQC used to realize the XOR function on quantum computers. It details the architecture of the VQC, including the choice of quantum gates and their roles in encoding nonlinear decision boundaries. This section also describes the encoding scheme for the inputs and outputs, the training procedure using parameter-shift gradients within the PennyLane framework, and the process of transferring the optimized parameters from the simulation to physical hardware. This comprehensive approach ensures effective learning and execution of the XOR mapping on a desktop quantum device, bridging theoretical design and practical deployment. Figure 1 illustrates the hardware platform and implementation workflow of the proposed QNN, highlighting the parameter transfer from simulation to the NMR-based quantum hardware and the subsequent measurement and tomography procedures. The NMR hardware consists of an RF coil that interacts with the sample, applies RF pulses, and senses the resulting signal. It is also equipped with environmental sensors and control systems that sense and maintain a stable operating temperature.

### 2.1. Variational Quantum Circuit Architecture

XOR is a function that classic linear models cannot learn, whereas VQC inherently possesses the ability to express nonlinearities [20]. In the VQC, the output is the measurement probability, where the measurement is performed on the second (readout) qubit of the two-qubit system, as shown in Equation (2):

(2) $$\begin{array}{c} p\left(1\right)=\mathrm{Tr}\;\left[\rho \left(\theta \right)\left(I⊗|1\rangle \langle 1|\right)\right]. \end{array}$$

Here, ρ(θ) denotes the two-qubit density matrix prepared by the variational circuit with parameters θ, *I* is the identity operator acting on the first qubit, and |1〉〈1| is the projector corresponding to measuring the second (readout) qubit in the computational basis.

Owing to the rotation, superposition, interference, and entanglement of the quantum state, the final measurement probability becomes a highly nonlinear function, as expressed in Equation (3):

(3) $$\begin{array}{ccc} p(1) & = & f\left(\theta \right)\cos^{2}\left(\cdots \right)+g\left(\theta \right)\sin^{2}\left(\cdots \right)\\ \; & \; & +h(\theta )\sin(\cdots )\cos(\cdots ). \end{array}$$

Therefore, VQC is suitable for imitating the nonlinear structures of neural networks.

**Figure 2:**
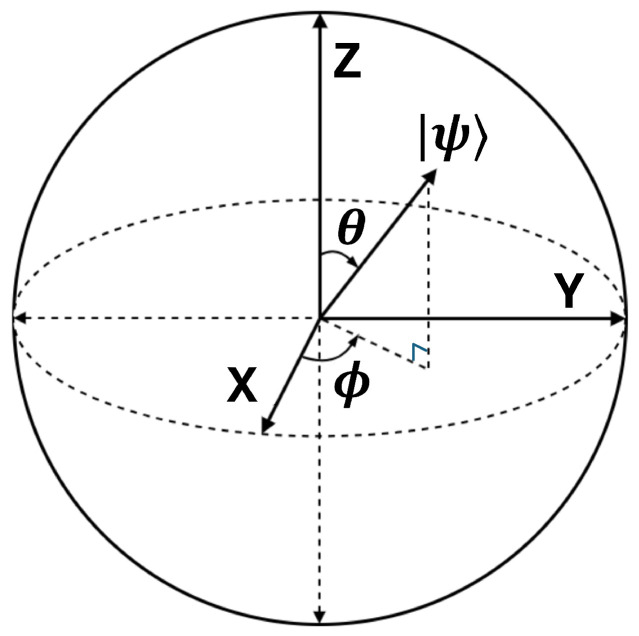
Bloch sphere representation of the qubit state.

Next, we choose $R_{y}\left(\theta \right)$ as the training parameter because $R_{y}\left(\theta \right)$ can be directly mapped to the “amplitude change” on the Bloch sphere, which is most likely to affect the output probability. As shown in Figure 2, on the Bloch sphere, the action of the Ry quantum gate is to rotate around the *y*-axis, and the same applies to $R_{x}$ and $R_{z}$ [21]. Because we ultimately measure the z-basis |0〉 and |1〉, $R_{y}\left(\theta \right)$ can directly change the amplitudes of |0〉 and |1〉, as shown in Equation (4):

(4) $$\begin{array}{ccc} R_{y}\left(\theta \right)|0\rangle \; & = & \cos\;\left(\frac{\theta }{2}\right)|0\rangle +\sin\;\left(\frac{\theta }{2}\right)|1\rangle . \end{array}$$

As a simple single-qubit example, the probability of measuring the outcome |1〉 after an $R_{y}\left(\theta \right)$ rotation is given by Equation (5):

(5) $$\begin{array}{ccc} p(1) & = & \sin^{2}\;\left(\frac{\theta }{2}\right). \end{array}$$

The result can be nonlinear, continuously differentiable, and directly related to the output probability.

Additionally, $R_{z}$ only alters the phase and does not change the Z-basis measurement probability; thus, it is not suitable as the primary training parameter. $R_{x}$ can also cause amplitude changes, but $R_{x}$ is highly sensitive to NISQ noise. $R_{y}$ is of crucial importance in the training of quantum neural networks because it can directly and continuously alter the measurement probability under the calculation of the basis measurement, thereby providing stable, differentiable and expressive parameter channels; in contrast, $R_{z}$ does not change the probability distribution under the same measurement basis and cannot effectively participate in the training. Therefore, $R_{y}$ is the most natural and compatible choice for the software. However, in terms of hardware, $R_{x}$ is superior. This is explained in the comparison section.

Because XOR is nonlinearly separable, it must rely on “interaction terms” to be expressed. In quantum circuits, entangling gates provide a mechanism for creating such interaction terms, making entanglement essential for the circuit to express the XOR function [22]. The truth table for the CNOT gate is presented in Table 2. In a 2-qubit VQC, CNOT is the only single-gate operation that can generate entanglement, whereas other quantum gates make hardware execution more difficult.

In the experiment, we have two channels, $Q_{0}$ and $Q_{1}$, to which $x_{0}$ and $x_{1}$ are the inputs. In this article, |0〉 and |1〉 represent the computational ground states defined by two distinguishable energy levels in a specific physical system, rather than abstract mathematical constructs. In experiments, quantum bits are first prepared in their ground state |0〉 through system initialization. This process typically corresponds to the natural ground state of a physical system or a stable state after active polarization. Subsequently, through controlled external driving (such as RF or microwave pulses), a transition between the two energy levels is induced under resonance conditions, thereby achieving the preparation of |0〉 to |1〉. This process has been realized on various physical platforms, including spin systems, quantum dot structures, and optical polarization systems. Therefore, |0〉 and |1〉 have clear physical correspondences and are achievable. Before entering the entanglement layer, the qubits are in the computational basis states |0〉/|1〉. While fixed basis states can already lead to entangled outputs under a CNOT operation, such inputs restrict the accessible quantum state space and limit the expressivity of the circuit. To introduce continuous degrees of freedom and enable a more flexible exploration of the quantum-state manifold, parameterized single-qubit rotations were applied prior to the entangling gate. These rotations do not create entanglement by themselves, but modulate the input state such that the subsequent entangling operation can explore a richer set of quantum correlations. Therefore, we need two $R_{y}$ gates to transform the input |0〉/|1〉 into a trainable state, determine the type of entanglement that the CNOT will generate, and thereby lay the foundation for nonlinearity. Subsequently, we can obtain the first quantum state as shown in Equation (6):

(6) $$\begin{array}{c} |\psi_{1}\rangle =R_{y}\left(\theta_{0}\right)|x_{0}\rangle ⊗R_{y}\left(\theta_{1}\right)|x_{1}\rangle . \end{array}$$

Here, $|x_{0}\rangle$ and $|x_{1}\rangle$ are the computational basis states encoding the classical XOR inputs, and $R_{y}\left(\theta \right)$ denotes a single-qubit rotation around the *y* axis with variational parameter θ.

This step creates the amplitudes $\cos\;\left(\frac{\theta }{2}\right)$, $\sin\;\left(\frac{\theta }{2}\right)$. After passing through the CNOT gate, the quantum state becomes complex, as shown in Equation (7):

(7) $$\begin{array}{c} |\psi_{2}\rangle =a|00\rangle +b|01\rangle +c|10\rangle +d|11\rangle . \end{array}$$

If measured directly at this point, it would lead to insufficient model expressiveness, inability to adjust the output, and failure to push the entangled quantum state to the four target points of the XOR. Therefore, the second layer of $R_{y}$ is required to “shape” the entangled quantum state. We add two more $R_{y}$ gates to adjust the measurement results, expand the model’s expression ability, achieve the final decision boundary, successfully converge to XOR, and obtain the final quantum state, Equation (8):

(8) $$\begin{array}{c} |\psi_{f}\rangle =\left(R_{y}\left(\theta_{2}\right)⊗R_{y}\left(\theta_{3}\right)\right)|\psi_{2}\rangle . \end{array}$$

**Figure 3:**
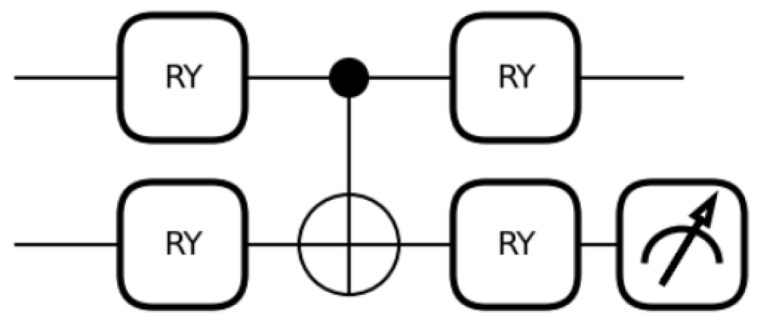
VQC with a *R_y_*-CNOT-*R_y_* structure.

Based on the above, we designed a VQC with a $R_{y}$-CNOT-$R_{y}$ structure, as shown in Figure 3.

### 2.2. XOR Input and Output Encoding Scheme

First, we set the training data *x* and *y*, with the input $x=\left(x_{0},x_{1}\right)\in {\left\{0,1\right\}}^{2}$ and target output $y=x_{0}⊕x_{1}$. In addition, we used ground-state encoding, which is simple, discrete, and hardware-friendly. Notably, the ground state of the quantum computer we use can only be |00〉; therefore, a Pauli-X gate needs to be applied to the corresponding qubit to flip |0〉 to |1〉. Subsequently, we measure the second qubit and return the *Z*-basis probability vector [p(0),p(1)]. The model output is defined as $\hat{y}\left(x\right)=p\left(1|x\right)$, that is, the probability of measuring |1〉 is taken as the “predicted XOR output.” Because the result of XOR is either 0 or 1, and the output of our VQC is the probability p(1∣x) of measuring 1, we hope that the final output of the model, which is the ”probability of measuring 1”, should be equal to the result of the XOR operation, Equation (9):

(9) $$\begin{array}{c} p\left(1|x_{0},x_{1}\right)\approx x_{0}⊕x_{1}. \end{array}$$

### 2.3. Training Procedure

As mentioned before, we need four $R_{y}$ gates, and we take the angles θ as the training objects. The initial circuit parameters were sampled from a uniform distribution over [0,2π), corresponding to a mean value of π and a variance of $\pi^{2}/3$. The first layer is $\theta_{0}$ and $\theta_{1}$, and the second layer is $\theta_{2}$ and $\theta_{3}$. Regarding the selection of the loss function, because the output of the quantum circuit is essentially an effective probability distribution, the mean square error (MSE) is smooth, continuous, and differentiable [23], suitable for small-scale VQC, and makes it easier for the VQC to successfully converge. The MSE is perfectly matched with the quantum probability output structure, and no additional transformation is required, as shown in Equation (10):

(10) $$\begin{array}{c} L\left(\theta \right)=\frac{1}{4}\sum_{\left(x,y\right)}{\left(p_{\theta }\left(1|x\right)-y\right)}^{2}. \end{array}$$

Traditional deep learning is based on backpropagation, but quantum circuits are unitary and cannot be directly backpropagated [24]. Therefore, we use PennyLane’s Gradient Descent Optimizer, Equation (11):

(11) $$\begin{array}{c} \frac{\partial f(\theta )}{\partial \theta }=\frac{f\;\left(\theta +\frac{\pi }{2}\right)-f\;\left(\theta -\frac{\pi }{2}\right)}{2}. \end{array}$$

This eliminates the need to derive gradients, write shift versions of circuits or implement backpropagation. It also enables automatic differentiation of all trainable parameters. Because there are only four sets of data for XOR, we adopted full-batch training (updating the parameters with all four samples at once). Thus, each step fully traverses the four training points, ensuring that the gradient is accurate and unbiased. Unlike mini-batches, there is no variance, and the update direction is more stable and closer to the true optimal direction. The basic update rule of the gradient descent algorithm [25] is given by Equation (12):

(12) $$\begin{array}{c} \theta \leftarrow \theta -\eta \frac{\partial L}{\partial \theta }. \end{array}$$

Here, θ are the four trainable angle parameters, the step size η (learning rate) for each update is 0.4, and $\frac{\partial L}{\partial \theta }$ is the partial derivative of the loss function with respect to the parameters. During the entire training process, the initial value θ is randomly selected from 0 to 2π. PennyLane automatically computed the gradients of all parameters through the parameter-shift rule, updated the parameters using gradient descent, and then returned the new θ and new loss. All experiments were performed with a fixed random seed (seed = 0) for parameter initialization to ensure reproducibility of the results. We set this loop to repeat 200 times in our study. Training was terminated when the loss function fell below $10^{-4}$, indicating convergence, or when the maximum number of training steps (200) was reached to avoid unnecessary over-optimization.

After training, we observed that the loss decreased rapidly and converged stably; the parameters remained stable without fluctuations, and the output probabilities matched the truth table of the XOR operation.

### 2.4. Parameter Transfer

We obtained four $R_{y}$ rotation angle parameters after training using the PennyLane library. These θ correspond to the angles of the ideal quantum gates. The gate model of the desktop quantum computer is a pulse model (NMR implementation), and the $R_{y}$ gate is essentially a rotation around the *y*-axis, corresponding to the RF pulse of the H-channel or P-channel, as shown in Equation (13):

(13) $$\begin{array}{c} R_{y}\left(\theta \right)\equiv \exp\;\left(-i\;\frac{\theta }{2}\;\sigma_{y}\right). \end{array}$$

In this desktop quantum computer, the rotation angle is determined by the product of the pulse amplitude and duration, as expressed in Equation (14).

(14) $$\begin{array}{c} \theta =\gamma B_{1}t_{p}. \end{array}$$

Here, γ represents the gyromagnetic ratio, $B_{1}$ is the RF field amplitude (fixed by the platform), and $t_{p}$ is the pulse duration (which can be adjusted). Therefore, in a desktop quantum computer, “setting the pulse width $t_{p}$” is equivalent to setting the rotation angle θ. The platform provided the calibrated duration $t_{\pi }$ of the π pulse; therefore, we can convert θ into the pulse width of each gate, as shown in Equation (15).

(15) $$\begin{array}{c} t_{p,i}=\left(\frac{\theta_{i}}{\pi }\right)t_{\pi }. \end{array}$$

We validated the transfer by running a pulse-only version of the circuit and confirming that the output probability distribution matched the simulated distribution within the experimental precision.

### 2.5. Tomography Procedure

Measurements are performed in the Pauli bases {X,Y,Z}, which form a complete operator basis for single-qubit state reconstruction, allowing the estimation of the expectation values 〈X〉, 〈Y〉, and 〈Z〉. Quantum hardware can only be measured on the computational basis (Z basis): {|0〉,|1〉}, so measuring X or Y essentially involves rotating the eigenstates of X or Y to the Z-basis. The eigenstates of X are $|+\rangle =\left(|0\rangle +|1\rangle \right)/\sqrt{2}$ and $|-\rangle =\left(|0\rangle -|1\rangle \right)/\sqrt{2}$. The Hadamard gate satisfies H|+〉=|0〉 and H|−〉=|1〉. That is, H→Zmeasurement≡Xmeasurement; The eigenstate of y is: $|+i\rangle =\left(|0\rangle +i|1\rangle \right)/\sqrt{2}$, $|-i\rangle =\left(|0\rangle -i|1\rangle \right)/\sqrt{2}$, through $S^{†}H|\pm i\rangle =|0\rangle ,|1\rangle$. Therefore, $S^{†}H|\pm i\rangle =|0\rangle ,|1\rangle$. The same measurement procedure is applied in both simulation and hardware experiments to ensure consistency.

For each measurement basis, the circuit is executed with *N* repeated shots to estimate the measurement outcome probabilities $p_{0}$ and $p_{1}$. The expectation values of the Pauli operators are then calculated using Equation (16).

(16) $$\begin{array}{c} \langle P\rangle =p_{0}-p_{1},\;P\in \left\{X,Y,Z\right\}. \end{array}$$

The single-qubit density matrix is reconstructed via linear inversion using the measured expectation values according to Equation (17).

(17) $$\begin{array}{c} \rho =\frac{1}{2}\left(I+\langle X\rangle X+\langle Y\rangle Y+\langle Z\rangle Z\right). \end{array}$$

The reconstructed density matrices are subsequently used for quantitative and qualitative analyses. State fidelity is evaluated to quantify the agreement between the simulated and hardware-executed quantum states. In addition, the expectation values obtained from tomography are used to visualize quantum states on the Bloch sphere. This enables a direct and consistent state-level comparison between simulation and hardware results after parameter transfer.

## 3. Experimental Setup

This section describes the simulation environment and physical quantum hardware platform used to implement and evaluate the quantum neural network for the XOR task. It details the specifications of desktop NMR quantum computers, including qubit types, operating frequencies, and control mechanisms. It also covers the calibration procedures essential for accurate pulse generation and system stability, as well as a step-by-step protocol for preparing the input states, executing the variational quantum circuit, and performing measurements. This comprehensive setup ensures that the theoretical design and training of the quantum circuit can be effectively translated into practical experiments on quantum hardware.

### 3.1. Simulation Environment

We used the Python programming language, PennyLane QML framework, and random seed settings. The computations were performed on an Intel i7 CPU with 16 GB RAM hardware, and multiple repeated experiments were conducted to perform full-state simulations of a 2-qubit quantum system.

### 3.2. Quantum Hardware Platform

The quantum computing platform that we adopted is an NMR desktop quantum computer, which features a nuclear magnetic resonance system with two nuclear spins (H1 and P31), [17]. Regarding the Larmor frequency, we set the frequency of the H channel to 27.12 MHz and that of the P channel to 10.98 MHz. The nuclear spins of H and P were controlled using a dual-channel RF pulse generator. Each channel can synthesize phase-coherent pulses with programmable amplitude, frequency, phase, and duration, enabling the implementation of precise single-qubit rotations and controlled operations on qubits. The gate operations specified in the experiment were compiled by the built-in pulse sequence compiler into low-level RF pulse instructions. The compiler automatically manages the pulse timing, channel synchronization, phase tracking across pulses, and insertion of J-coupling evolution intervals in the sequence. This abstraction layer enables the reliable execution of trained variational quantum circuits on hardware without requiring manual pulse engineering.

### 3.3. Pulse Calibration and Device Characterization

Before conducting the experiment, we performed a calibration. This can sense, detect, and correct system-level defects, thereby ensuring that subsequent series of quantum operations are completed with consistent pulse fidelity. The first step was Larmor frequency calibration. The system automatically detects the resonance frequencies of the H and P nuclear spins by sweeping the RF spectrum and identifying the peak responses. This ensured that all RF pulses were applied exactly at resonance, thereby minimizing the off-frequency errors.

The second step was to calibrate the pulse amplitude and duration. The system stores the optimized amplitudes and widths of the pulses for the two qubits in the control module and uses them to synthesize the arbitrary rotation angles required for the VQC. Calibration also aligns the relative phase of the RF channels, ensuring that single-qubit rotations are performed using stable-phase references. This prevents systematic phase drift during multistep pulse sequences.

### 3.4. Circuit Implementation Procedure

First, we prepared an input state. Because the system only has the |0〉 state, we can add an $R_{x}$ gate to obtain the |1〉 state. Subsequently, we input the training angle parameters obtained from the simulation into four Ry gates. After state preparation, the trained variational circuit is executed layer by layer. For each layer, the hardware applies the required single-qubit rotations, followed by an entangling operation mediated by the intrinsic J-coupling between the nuclear spins. Subsequently, the recorded data of channel Q1 were run and observed, and the experiment was repeated multiple times.

During execution on a desktop quantum computer, each run begins with the preparation of a classical input encoded in two nuclear spins. The system naturally initializes both qubits in |0〉, and states that require a population inversion are produced by applying an $R_{x}\left(\pi \right)$ pulse to the appropriate spin.

Once the input was determined, the variational circuit was obtained from the simulation in the order prescribed by the gate sequence. Single-qubit rotations were generated using previously calibrated RF pulses on the H and phosphorus (P) channels. The entangling step relies on the natural scalar J-coupling of the two spins, with the control system arranging the timing and evolution intervals such that the entire sequence remains well within the coherence time of the quantum device.

After the last operation, the state of output qubit Q1 was read using the standard NMR detection routine. For each input pattern, the measurement was repeated several times, and the resulting data were averaged to obtain a stable estimate of the output probability.

## 4. Simulation Results

This section presents the outcomes of the training and evaluation of the VQC designed to learn the XOR function. The analysis of the training convergence demonstrated rapid loss reduction and stable parameter optimization. The section then details the optimized rotation parameters and compares the model’s predicted outputs with the expected XOR truth table, highlighting minimal errors. Each experiment was repeated 3 times, and the results were averaged to reduce statistical fluctuations. Experimental results from the quantum hardware are also provided, including quantum state tomography data, fidelity, and purity metrics, which collectively confirm the successful implementation and high accuracy of the QNN on practical quantum hardware.

### 4.1. Training

**Figure 4:**
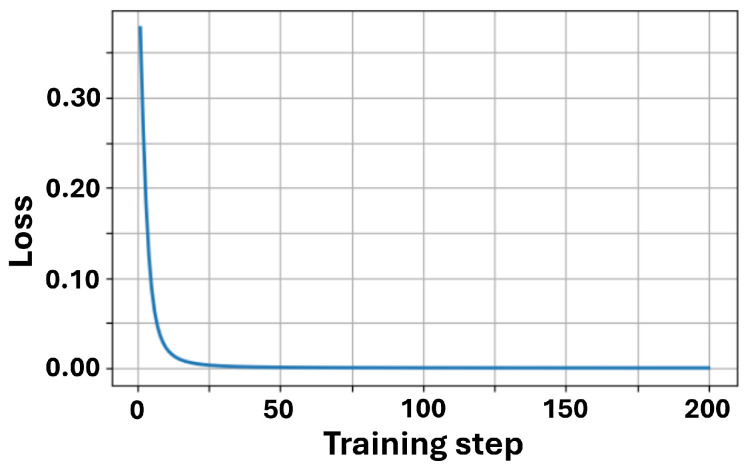
Training loss vs. steps.

We first examined the training convergence curve, as shown in Figure 4. From the simulation results, we obtained an initial loss of approximately 0.38 and a final loss of $4.06\times 10^{-5}$. The loss value dropped rapidly within the first 20 steps and stabilized by the 50th step, indicating that the VQC effectively learned the XOR mapping, which is consistent with our expectations.

As shown in Table 3, we obtained four $R_{y}$ rotation angle parameters after PennyLane training. The relatively large values of $\theta_{2}$ and $\theta_{3}$ indicate that the circuit relies on a substantial number of single-qubit rotations combined with entangled CNOT gates to form nonlinear decision boundaries, which represents an operation that is unattainable by classical single-layer perceptrons.

A comparative analysis between the model predictions and ground truth values, as presented in Table 4, demonstrates minimal error margins that align with our anticipated results.

**Figure 5:**
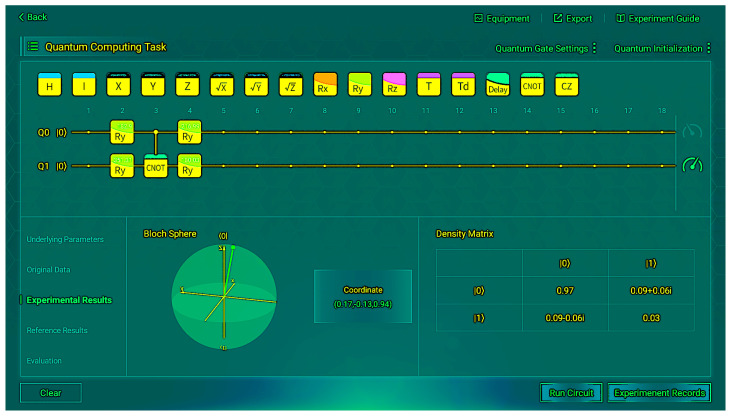
Experimental results (*R_y_*) for input |00〉.

**Figure 6:**
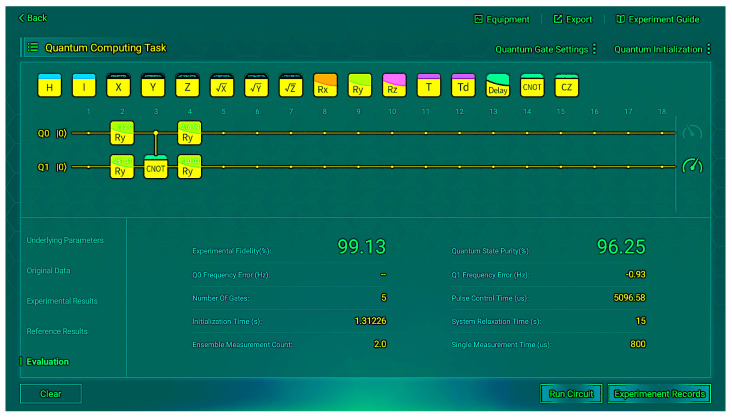
Evaluation metrics (*R_y_*) for input |00〉.

Through multiple experimental trials and subsequent averaging, we obtained a reliable comparative table of experimental data against the reference data, as shown in Table 5. We present some of the operation results from the measurements in Figure 5 and Figure 6. These figures depict the situation when the input is |00〉.

We found almost no difference between the experimental and reference states. When the input is |00〉, 97% of the time the correct output |0〉 can be obtained; when the input is |01〉, 98% of the time the correct output |1〉 can be obtained; when the input is |10〉, 98% of the time the correct output |1〉 can be obtained; when the input is |11〉, 95% of the time the correct output |0〉 can be obtained. Although there is some coherence in the non-diagonal, which indicates a small amount of noise, the impact is insignificant. This indicates that the final state of the real hardware is very close to the pure state of |0〉 or |1〉, and the density matrix verifies the correctness of the VQC structure.

Finally, we conducted multiple experiments and obtained the average values for fidelity and purity, as presented in Table 6. The results demonstrate that both fidelity and purity consistently maintain high percentages, indicating that the VQC successfully implements the quantum mapping of the XOR and operates stably on actual hardware.

### 4.2. Verifications

First, we investigate the relative sensitivity of the $R_{x}$- and $R_{y}$-based circuits under the considered noise levels. To this end, we repeat the training procedure using $R_{x}$ as the variational rotation and introduce additional noise sources into the QNN simulation to mimic generic NISQ-era imperfections. The employed noise setting is intended as a phenomenological model to probe noise sensitivity trends rather than a hardware-faithful quantitative noise characterization. The final loss values with and without noise are listed in Table 7. Based on these results, we define a degradation factor as the ratio between the final loss obtained with noise and that obtained in the noiseless case, as expressed in Equation (18):

(18) $$\begin{array}{ccc} D_{\mathrm{RY}} & = & \frac{0.00681}{4.06\times 10^{-5}}\approx 168,\\ D_{\mathrm{RX}} & = & \frac{0.00695}{1.57\times 10^{-5}}\approx 442. \end{array}$$

Under this definition, the degradation factor of the $R_{x}$ circuit is larger than that of the $R_{y}$ circuit, indicating a stronger degradation of the final loss under the considered noise setting. We note that the degradation factor serves as a coarse indicator of noise-induced performance degradation and does not fully capture training stability or decision-level correctness. A more comprehensive analysis incorporating additional metrics is left for future work.

**Figure 7:**
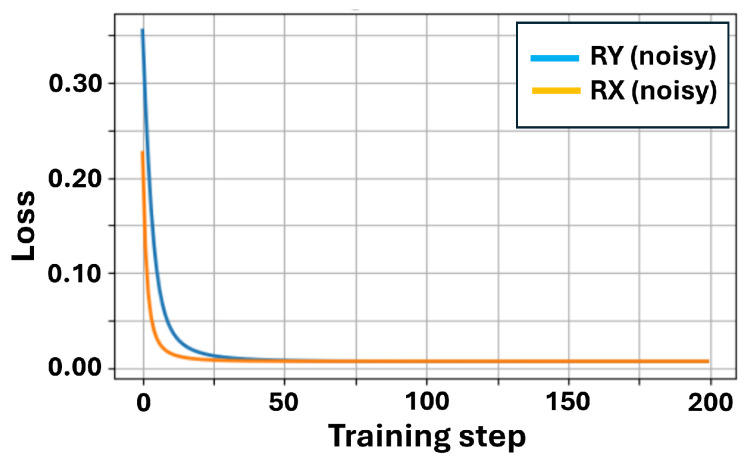
XOR training: RY vs. RX (with noise).

**Figure 8:**
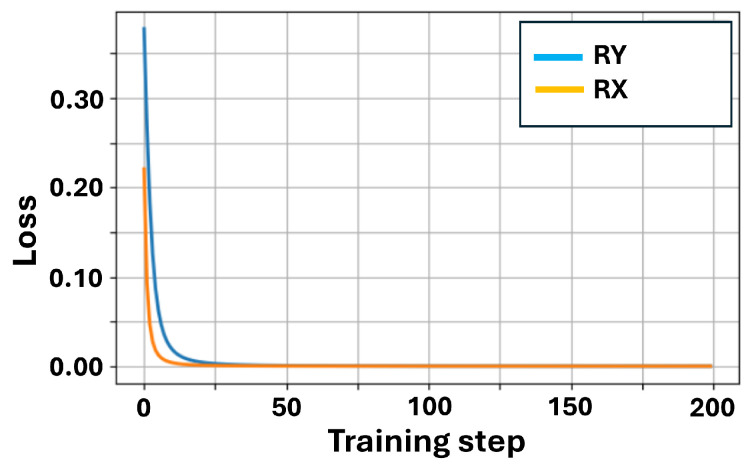
XOR training: RY vs. RX (noiseless).

From the training curves in Figure 7 and Figure 8, it can be observed that $R_{x}$ performs as well as $R_{y}$, with the loss rapidly decreasing to nearly zero within the first 50 steps. When $R_{y}$ rotates, $R_{y}\left(\theta \right)|0\rangle =\cos\frac{\theta }{2}|0\rangle +\sin\frac{\theta }{2}|1\rangle$, both coefficients are real numbers without phase, and it can easily generate values of p(1) ranging from 0 to 1. When $R_{x}$ rotates, $R_{x}\left(\theta \right)|0\rangle =\cos\frac{\theta }{2}|0\rangle -i\sin\frac{\theta }{2}|1\rangle$. At this point, the coefficient of |1〉 has an -i phase. However, noise can easily eliminate the -i phase, and our measurement was performed in the Z basis. p(1) is completely determined by the amplitude of the wave function and not the phase. Therefore, when $R_{x}$ rotates, the output is prone to bias towards |0〉, and the starting point of $R_{x}$ is lower than that of $R_{y}$.

However, the noise in actual hardware differs from that in NISQ. The main source of noise is the frequency drift along the z-axis, which results in better performance of $R_{x}$ than $R_{y}$. During detuning the noise, the operation becomes $U=e^{-i\epsilon Z}R_{X}\left(\theta \right)$ for $R_{x}$ and $U=e^{-i\epsilon Z}R_{Y}\left(\theta \right)$ for $R_{y}$. Then, the Baker–Campbell–Hausdorff (BCH) Expansion is performed. For $R_{x}$, this is expressed by Equation (19):

(19) $$\begin{array}{c} e^{-i\epsilon Z}R_{X}\left(\theta \right)=R_{X}\left(\theta \right)\;e^{-i\epsilon Z}\;e^{\epsilon \theta Y/2} \end{array}$$

It can be observed that the error term is a rotation around the Y-axis. This type of error does not change the main rotation axis but only the phase in the X-Y plane, which is relatively easy to compensate. For $R_{y}$, Equation (20):

(20) $$\begin{array}{c} e^{-i\epsilon Z}R_{Y}\left(\theta \right)=R_{Y}\left(\theta \right)\;e^{-i\epsilon Z}\;e^{-\epsilon \theta X/2} \end{array}$$

It can be observed that the error term is a rotation about the X-axis. This type of error directly alters the rotation axis. Originally rotating around Y, it rotates around the “Y + a small amount of X” axis, resulting in a skew.

Unfortunately, we cannot introduce exactly the same noise as that in the desktop quantum computer into the QNN because the detuning (frequency drift along the *Z*-axis) is a continuous effect, not a discrete $R_{z}$ operation. However, detuning can be continuously applied throughout the entire RF pulse period, continuously during free evolution, and still exists for phase accumulation during the readout. This cannot be fully simulated using a discrete gate.

**Figure 9:**
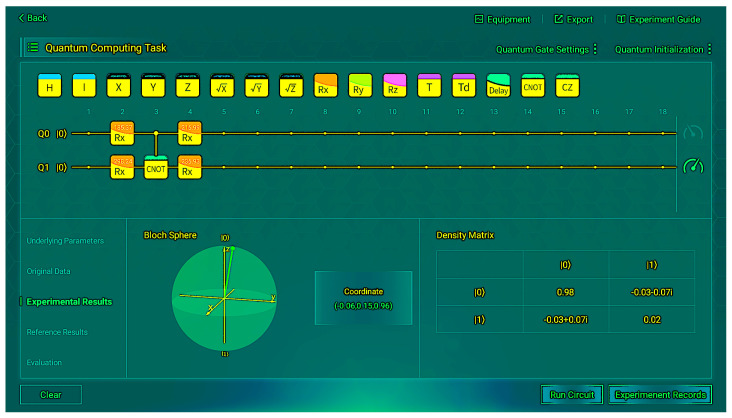
Experimental results (*R_x_*) for input |00〉.

**Figure 10:**
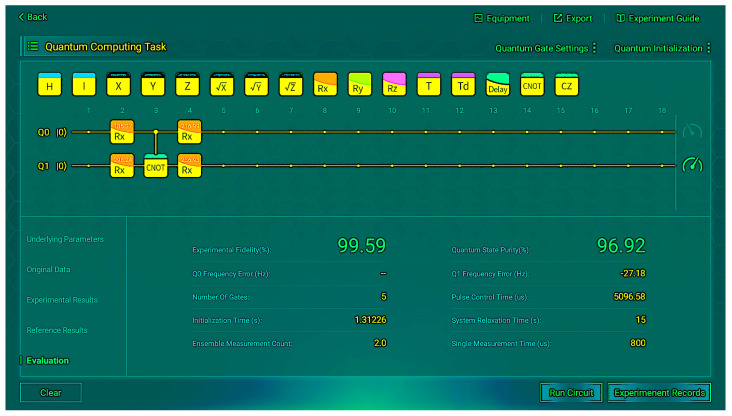
Evaluation results (*R_x_*) for input |00〉.

We then implemented the hardware system. The trained $R_{x}$ parameters are presented in Table 8. The measurement was then conducted on the desktop quantum computer, and we present some of the operation results of the measurements, as shown in Figure 9 and Figure 10. These figures depict the situation when the input is |00〉.

The final results are listed in Table 9. We can observe that $R_{x}$ can perform as well as $R_{y}$, and it is very close to the reference values.

We performed 10 shots per input to ensure statistical reliability of the results and recorded the final average experimental fidelity and quantum state purity, as shown in Table 10.

We compared the final average result of $R_{x}$ with that of $R_{y}$, as shown in Table 11. It can be observed that the performance of $R_{x}$ is superior, which is consistent with our previous reasoning.

We note that fidelity and purity are state-level metrics that characterize the similarity and mixedness of quantum states, respectively. While these metrics are useful for verifying state-level consistency between simulation and hardware, they do not directly quantify the task-level correctness of the learned XOR mapping, such as decision margins or classification outcomes under finite readout noise. In this work, fidelity and purity are therefore used to validate parameter transfer and state preparation, rather than to serve as direct measures of XOR decision performance.

## 5. Conclusions

Through this experiment, we demonstrated that QNN can learn nonlinear functions, such as XOR, effectively train VQC, and implement them on actual quantum hardware, achieving high fidelity and accuracy of the model. During the simulation phase, the VQC parameters converged to stability within 50 steps, and the results of the four experimental inputs were closely aligned with the reference outcomes. The overall average fidelity was 98.85%$\left(R_{y}\right)$ and 99.35%$\left(R_{x}\right)$, and the overall average purity was 95.16%$\left(R_{y}\right)$ and 97.43%$\left(R_{x}\right)$. These results show that a desktop quantum processor can perform QML workloads and that the VQC architecture can generate nonlinear mappings. Although the device size and hardware noise introduced certain limitations, and repeated runs may have exhibited small fluctuations, these factors did not alter the central conclusions of this study. In terms of algorithms, we can incorporate noise-aware training. This method enables the parameters to be adaptively adjusted to the slowly changing noise characteristics in the hardware (such as detuning and phase drift), thereby reducing the fluctuation of the results to a certain extent. In terms of hardware, we can enhance gate fidelity, improve frequency stability, and reduce detuning. These improvements can reduce the impact of noise on the evolution and measurement of quantum states at the source. The goal is to extend the research from XOR to multi-class quantum classification, explore different architectures, and conduct noise-aware training. If hardware improvements are achieved, the number of qubits can be increased.

## Figures and Tables

**Table 1:** Truth Table of the XOR function.

$x_{1}$	$x_{2}$	$\mathrm{XOR}(x_{1},x_{2})$
0	0	0
0	1	1
1	0	1
1	1	0

**Table 2:** Truth table of the CNOT gate.

Control	Target	Output Control	Output Target
0	0	0	0
0	1	0	1
1	0	1	1
1	1	1	0

**Table 3:** Optimized rotation parameters in radians and degrees (*R_y_*).

Parameter	Radian (Rad)	Degree (Deg)
$\theta_{0}$	3.192290	182.90
$\theta_{1}$	6.131568	351.31
$\theta_{2}$	3.787274	216.99
$\theta_{3}$	3.142093	180.03

**Table 4:** VQC performance on the XOR truth table.

Input	Target	VQC Output	Error
00	0	0.0064	0.0064
01	1	0.9936	0.0064
10	1	0.9963	0.0037
11	0	0.0037	0.0037

**Table 5:** Experimental and reference density matrices for all XOR inputs.

Input	Basis	Experimental $\rho_{\exp}$		Reference $\rho_{\mathrm{ref}}$	
		|0〉	|1〉	|0〉	|1〉
00	|0〉	0.97	0.09+0.06i	0.99	0.08
	|1〉	0.09−0.06i	0.03	0.08	0.01
01	|0〉	0.02	−0.10−0.05i	0.01	−0.08
	|1〉	−0.10+0.05i	0.98	−0.08	0.99
10	|0〉	0.02	0	0.01	0.08
	|1〉	0	0.98	0.08	0.99
11	|0〉	0.95	0.03i	0.99	−0.08
	|1〉	−0.03i	0.05	−0.08	0.01

**Table 6:** Fidelity and purity of four experimental runs (*R_y_*).

Input	Fidelity (%)	Purity (%)
00	97.90	91.43
01	98.84	95.17
10	99.54	97.80
11	99.13	96.25

**Table 7:** Final Loss Comparison Between RY and RX Circuits.

Circuit	Loss (No Noise)	Loss (With Noise)
RY	$4.06\times 10^{-5}$	$6.81\times 10^{-3}$
RX	$1.57\times 10^{-5}$	$6.95\times 10^{-3}$

**Table 8:** Optimized rotation parameters in radians and degrees (*R_x_*).

Parameter	Radian (Rad)	Degree (Deg)
$\theta_{0}$	3.235376	185.37
$\theta_{1}$	5.205278	298.24
$\theta_{2}$	3.787274	216.99
$\theta_{3}$	4.135212	236.93

**Table 9:** Experimental and reference density matrices for all XOR inputs (*R_x_*).

Input	Basis	Experimental $\rho_{\exp}$		Reference $\rho_{\mathrm{ref}}$	
		|0〉	|1〉	|0〉	|1〉
00	|0〉	0.98	−0.03−0.07i	1	−0.04i
	|1〉	−0.03+0.07i	0.02	0.04i	0
01	|0〉	0.01	0.12i	0	0.04i
	|1〉	−0.12i	0.99	−0.04i	1
10	|0〉	0.01	−0.10+0.02i	0	0.04i
	|1〉	−0.10−0.02i	0.99	−0.04i	1
11	|0〉	0.96	−0.02i	1	−0.04
	|1〉	0.02i	0.04	0.04i	0

**Table 10:** Fidelity and purity of four experimental runs (*R_x_*).

Input	Fidelity (%)	Purity (%)
00	99.59	96.92
01	99.58	100
10	99.34	100
11	98.89	92.78

**Table 11:** Average fidelity and purity of RX and RY circuits.

Metric	RX	RY
Average fidelity	99.35%	98.85%
Average purity	97.43%	95.16%

## Data Availability

Datasets are included in the manuscript.

## Abbreviations

The following abbreviations are used in this manuscript:

AI	Artificial Intelligence
MSE	Mean Squared Error
NISQ	Noisy Intermediate-Scale Quantum
NMR	Nuclear Magnetic Resonance
RF	Radio-Frequency
SLAEN	Supervised Learning Assisted by an Entangled Sensor Network
SVM	Support Vector Machine
QML	Quantum Machine Learning
QNN	Quantum Neural Network
VQC	Variational Quantum Circuit
XOR	Exclusive OR
